# Comparison of ^18^F-FDG, ^18^F-Fluoroacetate, and ^18^F-FEPPA for Imaging Liver Fibrosis in a Bile Duct-Ligated Rat Model

**DOI:** 10.1155/2021/7545284

**Published:** 2021-11-27

**Authors:** Chun-Yi Wu, Hsin-Hua Hsieh, Pei-An Chu, Wen-Hsiang Hong, Ting-Yu Chang, Chia-Fang Hsu, Siao-Ting Lin, Po-Hsun Su, Shin-Lei Peng

**Affiliations:** ^1^Department of Biomedical Imaging and Radiological Sciences, National Yang Ming Chiao Tung University, Taipei Branch, Taipei 112, Taiwan; ^2^Department of Biomedical Imaging and Radiological Science, China Medical University, Taichung 404, Taiwan

## Abstract

Developing sensitive diagnostic methods for a longitudinal evaluation of the status of liver fibrosis is a priority. This study is aimed at assessing the significance of longitudinal positron emission tomography (PET) imaging with ^18^F-labeling tracers for assessing liver fibrosis in a rat model with bile duct ligation (BDL). Twenty-one 6-week-old Sprague-Dawley male rats were used in this study. Longitudinal PET images using [^18^F]N-2-(2-fluoroethoxy)benzyl)-N-(4-phenoxypyridin-3-yl)acetamide ([^18^F]FEPPA) (*n* = 3), [^18^F]fluoroacetate ([^18^F]FAc) (*n* = 3), and 18F-fluoro-2-deoxy-D-glucose ([^18^F]FDG) (*n* = 3) were obtained at 0, 1, and 2 weeks after BDL. Biochemical assays, histological assays, immunohistochemical staining assays, and next generation sequencing analyses were also performed at 0 (*n* = 3), 1 (*n* = 3), 2 (*n* = 3), and 3 (*n* = 3) weeks after BDL, which demonstrated the severe damage in rat livers after BDL. Regarding [^18^F]FEPPA and [^18^F]FDG, there was a significantly higher uptake in the liver after BDL (both *P* < 0.05), which lasted until week 2. However, the uptake of [^18^F]FAc in the liver was not significantly different before and after BDL (*P* = 0.28). Collectively, both [^18^F]FEPPA and [^18^F]FDG can serve as sensitive probes for detecting the liver fibrosis. However, [^18^F]FAc is not recommended to diagnose liver fibrosis.

## 1. Introduction

Nonalcoholic fatty liver disease (NAFLD), including nonalcoholic fatty liver and nonalcoholic steatohepatitis (NASH), is a global health concern, with a rising prevalence in Asia [1]. As NASH may irreversibly progress from fibrosis to cirrhosis, the accurate and rapid assessment of liver condition plays a crucial role in formulating effective therapeutic plans. Liver biopsy has been considered the gold standard for the assessment of fibrosis. However, it is challenging for all patients to accept it due to its invasiveness and potential complications. Therefore, developing the sensitive diagnostic methods for determining the optimal therapeutic strategy and longitudinally monitoring the prognosis is on the list of priority [2].

Translocator protein (18 kDa) (TSPO), known as peripheral benzodiazepine receptor, is located on the outer membrane of mitochondria and is expressed in various peripheral organs but relatively low in the normal liver [3]. The liver of NASH patients has increased mitochondrial mass and biosynthesis due to adaptive stress response [4]. Xie et al. applied N-benzyl-N-methyl-2-[7,8-dihydro-7-(2-[^18^F]fluoroethyl)-8-oxo-2-phenyl-9H-purin-9-yl]acetamide ([^18^F]FEDAC), a specific positron emission tomography (PET) radiotracer for TSPO imaging, to quantify the expression level of TSPO in a methionine- and choline-deficient diet-fed mouse model and found that the uptake of [^18^F]FEDAC significantly elevated from simple steatosis to the NASH state [5]. Moreover, Hatori et al. indicated that the elevated accumulation of [^18^F]FEDAC in a chronic carbon tetrachloride-treated fibrotic liver was noticed when compared to controls [6]. On the other hand, TSPO is increased during the inflammation process, which is considered a common feature observed in chronic liver diseases [7]. Huang et al. demonstrated that the enhanced TSPO expression was accompanied by the higher uptake of [^18^F]N-fluoroacetyl-N-(2,5-dimethoxybenzyl)-2-phenoxyaniline ([^18^F]PBR06), another TSPO-target radiotracer in PET imaging, in the liver of bile duct-ligated rats within 4 weeks as compared to the control group [8]. Moreover, hepatic stellate cells (HSCs) are activated by inflammation-associated growth factors or cytokines to produce extracellular matrix during fibrogenesis, and the amount of these TSPO-expressing transformed HSCs and macrophages increased with the progression of fibrosis [9]. [^18^F]N-2-(2-fluoroethoxy)benzyl)-N-(4-phenoxypyridin-3-yl)acetamide ([^18^F]FEPPA), another specific ligand with a high affinity for TSPO, has been used as a useful biomarker for neuroinflammation [10] and breast cancer [11]. However, until date, little information is available on the application of [^18^F]FEPPA in evaluating liver fibrosis.

Oxidative stress has been considered another main contributor to liver injury and NAFLD pathogenesis [12]. [^18^F]fluoroacetate ([^18^F]FAc) has been developed to measure oxidative metabolism. Early work done by Ponde et al. demonstrated that [^18^F]FAc could be a useful tracer for the detection of prostate tumors [13]. However, the contradicting findings reported by Ho et al. have shown that [^18^F]FAc is not suitable to be recommended for evaluating hepatocellular carcinoma (HCC) and the consequent metastases [14]. As existing literature regarding the ability of [^18^F]FAc to detect the oxidative stress-related diseases remains controversial, the question whether [^18^F]FAc can be applied to detect the hepatic fibrosis is intriguing and warrants further investigation; however, no study to date is aimed at filling this gap.

This study is aimed at assessing the feasibility of [^18^F]FEPPA and [^18^F]FAc for the noninvasive evaluation of hepatic fibrosis status. The most commonly used radiocompound, ^18^F-fluoro-2-deoxy-D-glucose ([^18^F]FDG), was also employed for comparisons. Extending from the previous PET studies, the longitudinal experiment design in this study may advance our understanding in the role of molecular imaging in hepatic fibrosis.

## 2. Materials and Methods

### 2.1. Materials

The 4-0 suture was purchased from UNIK SURGICAL SUTURES MFG. CO., LTD. (New Taipei City, Taiwan). The Picro Sirius Red kit was purchased from Abcam (ab150681, Cambridge, UK). The FEPPA precursor (no. TEPP-90-0005) was purchased from Huayi Isotope Company (Shanghai, China). The [^18^F]FDG was kindly provided by Taipei Veterans General Hospital (Taipei, Taiwan). All other chemicals were purchased from Sigma-Aldrich Co. (St. Louis, MO, USA). GENEzol TriRNA Pure kit was purchased from Geneaid Biotech Ltd. (New Taipei City, Taiwan). Primary anti-GLUT1 (ab115730) and anti-TSPO (ab109497) antibodies were purchased from Abcam (Cambridge, UK). The secondary antibody was purchased from Thermo Fisher Scientific (New York, USA). The mounting medium was purchased from Abcam (ab236466, Cambridge, UK).

### 2.2. Animal Model

A total of 21 6-week-old Sprague-Dawley male rats were maintained at a stationary temperature and controlled humidity chambers. The rats were randomly assigned to seven groups of three rats each. Three groups served as longitudinal PET scanning groups (imaging group) for the three different types of radiotracers, respectively. In each imaging group, only one [^18^F]-tracer scan was performed longitudinally at 0, 1, and 2 weeks after bile duct ligation (BDL). The other three groups were used for serum analysis and histopathology assay (biochemical group) at 0, 1, and 2 weeks after BDL, respectively. The final group was used for additional Sirius red staining for the evaluation of liver fibrosis at 3 weeks after BDL.

**Figure 1:**
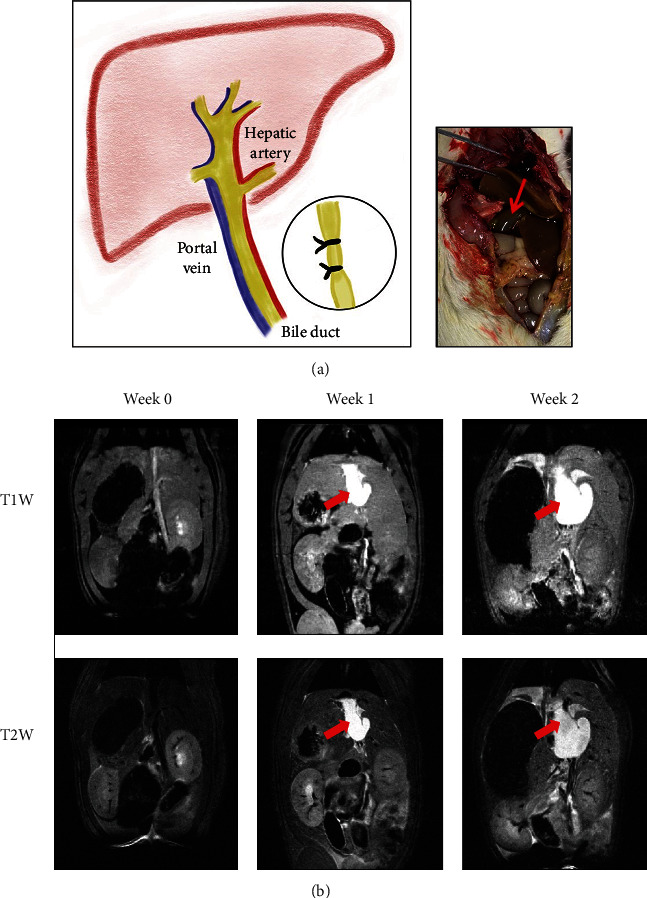
An example of bile duct ligation (BDL) in a rat model. (a) The scheme of bile duct ligation for inducing liver fibrosis. (b) The magnetic resonance images of a representative rat before and after BDL. The red arrows indicate the dilated bile ducts.

The BDL procedure was performed as described previously [15]. Briefly, the rats were maintained under anesthesia by letting them inhale a mixture of isoflurane (ISO) and oxygen (5% ISO for induction and 2% for maintenance). After sterilizing the shaved abdominal skin, a 2 cm middle incision was made to expose the surgical area. The portal vein and hepatic artery were carefully separated from the bile ducts. A 5-0 suture was placed around the bile duct and secured with a surgical knot. A second knot was made near the first ligation site to ensure the obstruction (Figure 1(a)). This protocol induced a high yield of cirrhosis in rats with morphological changes that were similar to those observed in human biliary cirrhosis [16]. All animal experiments were approved by the Local Animal Experimental Ethics Committee (CMU IACUC No. 2020-140 and NYCU IACUC No. 1100323).

### 2.3. Biochemical Assays

Animals in the biochemical groups were euthanized at weeks 0, 1, and 2 after BDL. Blood (~1 mL) was collected from the rats in each group. The blood was kept at 25°C for 2 h, followed by centrifugation at 12000 rpm for 10 min to obtain the sera. The biochemical indicators of liver function, including alanine aminotransferase (ALT), alkaline phosphatase (ALP), gamma-glutamyl transferase (GGT), and albumin levels, were measured using corresponding kits.

### 2.4. Histological Assay of Liver Fibrosis

The level of fibrosis was determined using Sirius red staining. The rats were sacrificed by perfusion with normal saline. The livers were fixed with 4% paraformaldehyde and embedded in paraffin. The 5 *μ*m slides were incubated with the Picro Sirius Red kit (ab150681, Abcam) for 30 min at 25°C to visualize collagen, while the nuclei were stained with hematoxylin. Images were captured using a bright-field microscope (BX61, Olympus, Japan). Quantification of the Sirius red area was performed using ImageJ (version 1.53j).

### 2.5. Next-Generation Sequencing (NGS) Analysis

Total ribonucleic acid (RNA) was extracted from the frozen liver using the Genezol TriRNA pure kit. The liver (<50 mg) was dissected and added to a 2 mL Eppendorf tub containing 700 *μ*L of Genezol reagent and kept at 25°C for 5 min. The mixture was centrifuged at 12,000 g for 1 min to separate the cell debris from the extract. Absolute ethanol was added to the extract (1 : 1, vol/vol), and the mixture was passed through the RB column for RNA binding. The column was sequentially eluted with prewash and wash buffers. Finally, the column was eluted with RNAase-free water to obtain the RNA. The mRNA concentration was evaluated using Nanodrop (ND-1000, NanoDrop Technologies Inc., USA). The NGS analysis was commissioned by BIOTOOLS Co., Ltd. (New Taipei City, Taiwan).

### 2.6. Preparation of [^18^F]FDG, [^18^F]FAc, and [^18^F]FEPPA

Commercialized [^18^F]FDG was prepared by a local hospital. The protocols for the production of [^18^F]FAc and [^18^F]FEPPA were basically based on the method published by Liu et al. [17] and Berroteran-Infante et al. [18], respectively. Both [^18^F]FAc and [^18^F]FEPPA had a radiochemical purity > 95%.

### 2.7. Animal PET/Magnetic Resonance Imaging (MRI)

An animal 7T micro-PETMR Inline (Bruker, Rheinstetten, Germany) was used for the PET studies. For animals in the three imaging groups, PET scans were longitudinally performed at weeks 0, 1, and 2 after BDL. The average weights of all animals in every week were 201.7 ± 7.6, 248.3 ± 7.6, and 315.0 ± 10.0 grams, respectively. Three different radiotracers (approximately 7.4 MBq) were injected via the tail vein for imaging. The specific activity at the first injection time was approximately 25 kBq/nmol (FDG), 15 kBq/nmol (FAc), and 5 MBq/nmol (FEPPA). Following the 60 min uptake of [^18^F]FDG and [^18^F]FAc, PET imaging was performed for 20 min, and [^18^F]FEPPA imaging [19] was performed for 30 min after 30 min uptake. All recorded PET images were used for analysis. Both T1-weighted (T1W) and T2-weighted (T2W) MR images were performed to determine the anatomical structure of the liver. The scanning sequences for T1W images were as follows: repetition time/echo time = 594 ms/2.7 ms, number of average = 2, spatial resolution = 0.36 × 0.42 × 1 mm^3^, and number of slices = 50. The scanning sequences for T2W images were as follows: repetition time/echo time = 4164 ms/24.7 ms, number of average = 4, spatial resolution = 0.22 × 0.25 × 1 mm^3^, and number of slices = 50. Heart and breath rates were recorded during the entire experiment, and body temperature was maintained using a warm water blanket.

**Figure 2:**
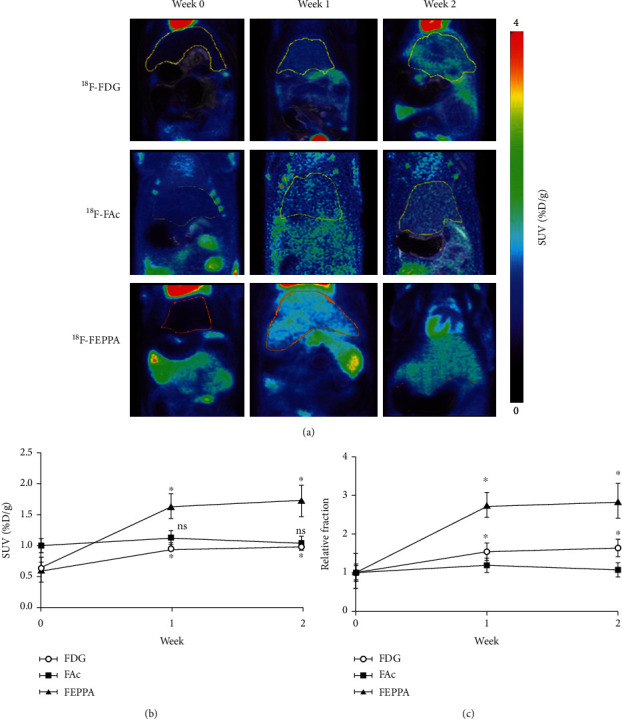
Accumulation of radioactivity in rats before and after bile duct ligation (BDL). (a) PET-MRI fusion images of representative rats before and after BDL. The solid lines on the PET-MRI fusion images indicate manually drawn liver ROIs. (b) The comparisons of the standard uptake value (SUV) of radioactivity at 0, 1, and 2 weeks after BDL. (c) The relative fraction of SUV compared to that at week 0 for each radiotracer. Data are presented as mean ± standard deviation. ^∗^Significantly different when compared to that before BDL.

The PET images were reconstructed using the three-dimensional ordered-subset expectation maximization method in the acquisition workplace. The PET images were reconstructed using the three-dimensional ordered-subset expectation maximization method in the acquisition workplace. All PET images were first coregistered to T2W MR images. For the analysis of the regions of interest (ROIs), the liver masks were delineated based on MR images. The generated liver masks were then applied to PET images. Examples of liver ROIs are shown in Figure 2(a). The regional radioactivity concentrations (KBq/c.c) of [^18^F]FEPPA, [^18^F]FAc, and [^18^F]FDG were estimated based on the mean pixel values within the liver regions. The radioactivity uptake in the liver was decay-corrected to the injection time and expressed as the standard uptake value (SUV) by dividing the radioactivity concentration by the whole-body concentration of the injected radioactivity. The radioactivity accumulation of each radiotracer in the liver was quantified using AMIDE (version 1.0.5).

### 2.8. Immunohistochemical Staining Assays

Animals in the biochemical groups were euthanized at weeks 0, 1, and 2 after BDL. The liver was excised, fixed with 4% paraformaldehyde at 25°C overnight, and then embedded in paraffin. The samples were cut into 5 *μ*m thick slices, which were sequentially soaked in xylene for 30 min at 25°C, absolute ethanol for 5 min at 25°C, and then in 0.01 M sodium citrate buffer (pH 6.0) at 95°C for 1 h. After rinsing with phosphate buffer solution (PBS), the slices were blocked with horse serum for 1 h at 25°C. The sections were incubated with primary anti-GLUT1 and anti-TSPO antibodies (1 : 100) at 25°C for 2 h. After the bioreaction, the sections were incubated with fluorophore-containing goat anti-rabbit immunoglobulin G secondary antibody (1 : 200) at 25°C for 1 h, followed by washing with PBS. After rinsing with PBS, the sections were mounted with a diaminobenzidine-containing mounting medium. The images were visualized using fluorescence microscope (BX61, Olympus, Tokyo, Japan).

### 2.9. Statistical Analysis

Data are expressed as mean ± standard deviation. To compare the biochemical data at different time points after BDL, the one-way analysis of variance (ANOVA) test was performed for ALT, ALP, GGT, and albumin results. To compare the uptake of radiotracers in the liver among the longitudinal PET scans, a one-way ANOVA with repeated measures was performed for the SUV. If the effect was observed in the ANOVA analysis, a post hoc Tukey's honestly significant difference test was employed. A *P* < 0.05 was considered statistically significant.

## 3. Results

### 3.1. MR Images after BDL

The MR images obtained using the T1W and T2W sequences before and after BDL are shown in Figure 1(b). The signal intensities of liver for T1W images at weeks 0, 1, and 2 were 12021.8 ± 376.2, 17367.9 ± 1007.3, and 15208.9 ± 82.9, respectively. The signal intensities of live for T2W images at weeks 0, 1, and 2 were 36.2 ± 1.2, 61.5 ± 1.6, and 62.8 ± 4.1, respectively. The common bile duct was significantly dilated after BDL on MR images, and the bile duct appeared hyperintense after BDL surgery on both T1W and T2W imaging, suggesting that the increased T1 and T2 values were associated with successful establishment of liver fibrosis [20].

### 3.2. Liver Function in the Serum

Significant cholestasis was observed because of the blocked flow of bile, which was caused by ligation. The biochemical data for ALT, ALP, GGT, and albumin levels are listed in Table 1. As presented, the levels of ALT, ALP, and GGT increased significantly after BDL (all *P* < 0.05) and peaked in week 1, reaching the plateau until week 2. The levels of ALT, ALP, and GGT were not significantly different between weeks 1 and 2. The albumin level did not change significantly after BDL.

### 3.3. The Levels of Liver Fibrosis in the Animal Model

**Figure 3:**
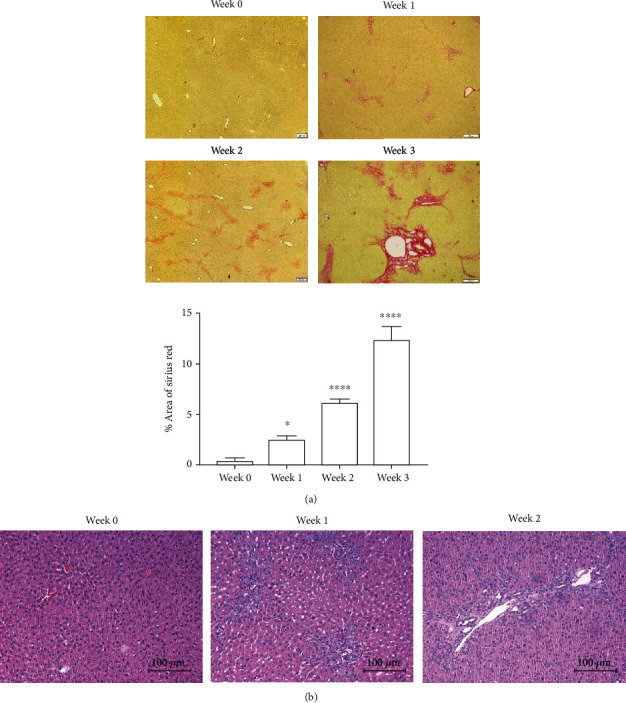
Histological assay of liver fibrosis: (a) Sirius red staining; (b) hematoxylin and eosin staining. ^∗^*P* < 0.05; ^∗∗^*P* < 0.01; ^∗∗∗^*P* < 0.001.

Following BDL, the apparent distribution of collagen was observed at weeks 1 and 2 (Figure 3). Three weeks after BDL, extensive crosslinks indicated that the liver was at the stage of cirrhosis. These results confirmed the deposition of extracellular matrix after BDL and indicated that ligated rats could serve as an appropriate animal model for further experiments. We also observed the expression of broken proteins and fibrils in hematoxylin and eosin stained samples with time after BDL (Figure 3(c)).

### 3.4. Next-Generation Sequencing (NGS) Analysis

**Figure 4:**
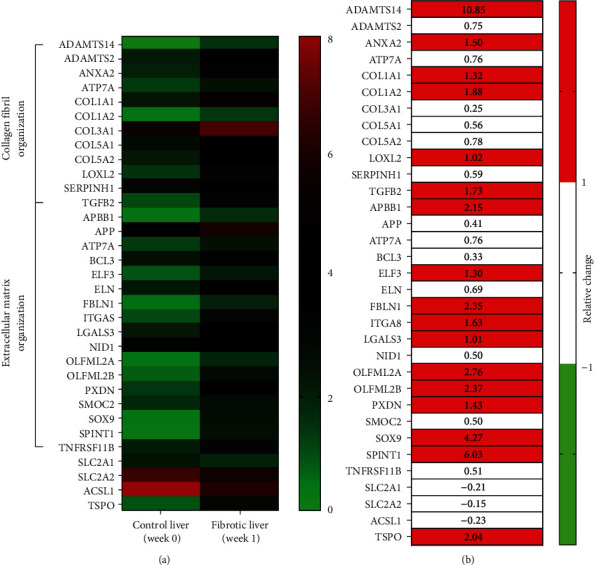
The next-generation sequencing analysis. (a) The heat map revealed the distinct ribonucleic acid (RNA) expression profiles between control (week 0) and fibrotic liver (week 1). The color bar indicates the range of expression values from 0 (green) to 8 (red). (b) The difference between normal and fibrotic livers. The color bar indicates the relative change more than 1 (red) or less than 1 (green) for the fibrotic liver with respect to the control liver ((fibrotic − control)/control). The list of abbreviations is in supplementation information (Table S1).

In the NGS analysis, we found that the levels of RNAs involved in glucose transport, TSPO expression, and fatty acid metabolism were significantly changed at weeks 1 after BDL when compared with those at week 0 (Figure 4(a)). The NGS data also confirmed that the RNAs associated with collagen fibril organization and extracellular matrix production were increased after the onset of fibrosis (Figure 4(b)). Specifically, the TSPO level increased in the fibrotic liver. The opposite result, i.e., control liver having higher expression values than the fibrotic liver, was not detected.

### 3.5. PET Imaging

**Figure 5:**
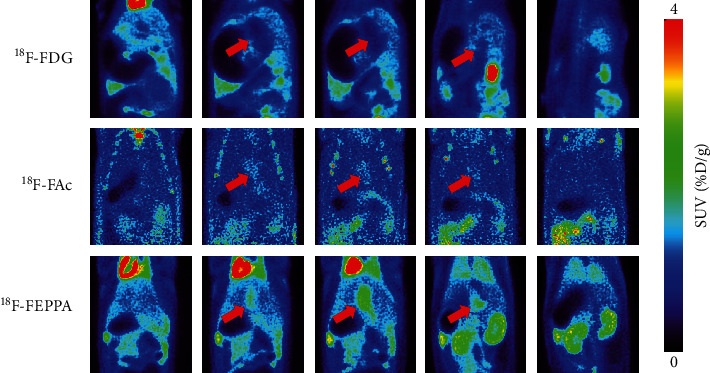
Positron emission tomography images at different slice locations 2 weeks after bile duct ligation. The red arrows indicate the dilated bile ducts.

Representative coronal PET-MRI fusion images and quantified liver uptake of the three different radiotracers before and after BDL are displayed in Figures 2(a) and 2(b), respectively. Typical ROIs are also shown. The liver uptake of [^18^F]FEPPA at 1 week postligation was almost 2.5-fold higher than that before BDL, while the increment of [^18^F]FDG was only 1.5-fold (both *P* < 0.01) (Figure 2(c)). Both [^18^F]FEPPA and [^18^F]FDG liver uptake reached a plateau at 1 week after ligation. However, there was no significant difference in [^18^F]FAc accumulation in liver among the three sets of PET images before and after BDL (*P* = 0.16). These results suggested that [^18^F]FEPPA may be a promising probe for the detection of liver fibrosis because of its relatively high sensitivity. PET images from representative rats at different slice locations 2 weeks after BDL are shown in Figure 5. The heterogeneity in radiotracer uptake in liver suggests that fibrosis is a heterogeneously distributed lesion by nature [21]. These images also showed a significantly dilated bile duct 2 weeks after BDL. In particular, the bile duct had a lower uptake of [^18^F]FDG but a higher uptake of [^18^F]FEPPA than that in the liver.

### 3.6. IHC Staining

**Figure 6:**
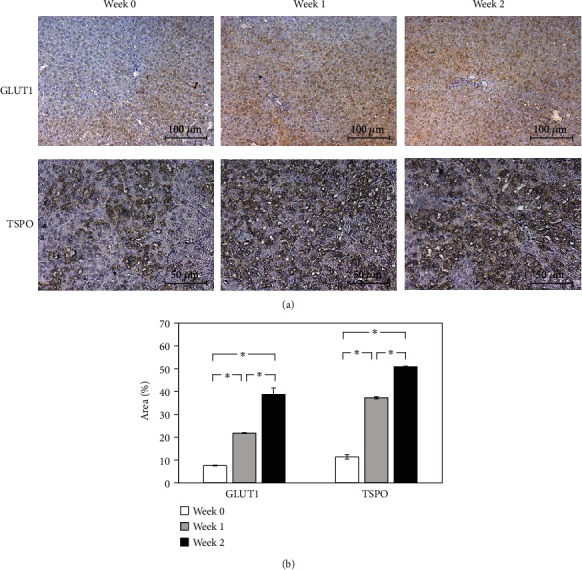
Immunohistochemistry (IHC) staining of livers for glucose transporter 1 (GLUT1) and anti-translocator protein (TSPO). (a) IHC images before and after bile duct ligation. (b) Quantitative analysis of the expression in the IHC images. ^∗^*P* < 0.05.

IHC staining demonstrated elevated expression levels of GLUT-1 and TSPO in the fibrotic livers (Figure 6). IHC showed the increased TSPO expression in the hepatic lobules around the bile duct, which correlated with the results of [^18^F]FEPPA imaging.

## 4. Discussion

Liver fibrosis, resulting from the accumulation of ECM collagens, is a wound healing response to chronic hepatic injury. However, this process irreversibly changes the original hepatic architecture because of continuous deposition of collagen and is accompanied by high mortality. A longitudinal monitoring procedure that is reliable and reproducible in clinical practice is desirable. To the best of our knowledge, this is the first study to perform longitudinal PET scanning at the animal model of hepatic fibrosis. Our results showed that both [^18^F]FEPPA and [^18^F]FDG have the advantages of detecting fibrosis in rats that received BDL, and both radiotracers demonstrated reproducibility over time. However, we found no evidence that [^18^F]FAc could be a potential radiotracer for PET evaluation of fibrosis in an animal model.

Several human studies have compared the uptake of [^18^F]FDG in the liver parenchyma between healthy individuals and patients with fibrosis. The existing reports in the literature show contradictory findings, which remain controversial. Hernandez-Martinez et al. showed that [^18^F]FDG accumulation was significantly reduced in liver fibrosis/cirrhosis group than that in the control group [22]. In patients with NASH, the [^18^F]FDG assessed using the SUV analysis showed little promise [23]. Nevertheless, the increased [^18^F]FDG update in liver fibrosis observed in this study is in general agreement with the findings of the human study reported by Verloh et al. [24] and the animal study reported by Su et al. [25]. With BDL, GLUT-1 levels quantified by IHC demonstrated increased expression in this study (Figure 5). There is evidence that a high glucose metabolism accompanied with increased [^18^F]FDG update in the liver parenchyma is linked with the overexpression of GLUT-1 [26]. The elevated uptake observed in our [^18^F]FDG study suggests that the fibrotic process is associated with GLUT-1 overexpression and can be assessed by noninvasive [^18^F]FDG PET.

TSPO has recently been considered as a useful biomarker of liver fibrosis [6, 8], and the increased mRNA expression level of TSPO in rats with BDL was also confirmed in this study (Figure 4). Previous studies have suggested that radiotracers, such as [^18^F]FEDAC [6] and [^18^F]PBR06 [8], can monitor liver fibrosis by assessing the expression levels of TSPO. [^18^F]FEPPA is another promising ligand for TSPO; however, the information obtained using [^18^F]FEPPA for evaluating liver fibrosis is missing. To our knowledge, this is the first study to evaluate liver fibrosis using [^18^F]FEPPA. We showed that [^18^F]FEPPA is highly sensitive to liver fibrosis, as demonstrated by a 2.5-fold uptake in the liver 1 week after BDL when compared to that before BDL. For [^18^F]FEDAC [6] and [^18^F]PBR068 [8], however, the magnitudes of the increased uptake after BDL were less than 50%. This discrepancy may be explained by the methodological factors. With respect to the cross-sectional design in the above studies [6, 8], the experimental setting of the longitudinal PET scanning before and after the onset of liver fibrosis in this study can provide a direct comparison of disease progression without interference from intersubject variations. Moreover, they acquired PET data immediately following tracer administration with an acquisition time of 30 min. Meanwhile, in our study, [^18^F]FEPPA imaging was performed for 30 min, after 30 min uptake. Comparisons of different scan durations in PET studies are ongoing and needed, and existing studies have suggested that imaging protocols have a significant effect on image quality [27, 28]. As we are only at an early stage in our attempt to understand the capability of [^18^F]FEPPA for TSPO imaging in evaluating liver fibrosis, optimized imaging protocols with regard to the scan duration or even administered dose will be the focus of future studies.

SUV is the most commonly used index for measuring radiotracer uptake in both clinical and preclinical studies. Our results showed that, after the onset of liver fibrosis, the magnitude of increased SUV was higher for [^18^F]FEPPA than for [^18^F]FDG, which may imply that [^18^F]FEPPA is more sensitive in detecting fibrosis. [^18^F]FDG is a hydrophilic solute that does not enter fat droplets in hepatocytes, resulting in a relatively lower uptake in the bile duct (Figure 5). The dilution effect of hepatic fat may have resulted in the heterogeneity of [^18^F]FDG uptake across the liver (Figures 2(a) and 5) and reduced SUV quantification [29]. On the other hand, TSPO PET ligands such as [^18^F]FEPPA showed outstanding properties regarding lipophilicity [18]. Accordingly, correcting the raw SUV by accounting for the proportion of the liver occupied by fat has been proposed to precisely quantify [^18^F]FDG uptake in the liver [30]. However, due to the limitation of the equipment, measuring fat proportion was not feasible in the present study, and we have only reported the results of the SUV quantification in order to be consistent with the existing literature. To determine whether differences in sensitivity in terms of fibrosis detection between [^18^F]FEPPA and [^18^F]FDG are due to interference from hepatic fat, further studies combining with fat quantification are highly recommended.

Radiolabeled acetates, such as [^11^C]acetate, have been recognized for years as useful tracers for measuring oxidative metabolism, and the use of [^11^C]acetate in the diagnosis of HCC is of equal importance [31, 32]. [^11^C]acetate can be activated and trapped as ^11^C-acetyl- coenzyme A (CoA) by the enzyme acetyl-CoA synthetase (ACS). The ACS family includes ACS1 and ACS2. Among these proteins, ACS1 is located in the cytosol. Cells with high lipogenic activity, such as the liver cells, often express higher levels of ACS1 and therefore trap [^11^C]acetate [33]. However, the short half-life of C-11 (20.4 min) has limited its widespread use. The fluorinated analogue, [^18^F]FAc, which has a longer half-life (110 min) than [^11^C]acetate, was recently proposed as a potential surrogate for assessing oxidative metabolism. Nevertheless, [^18^F]FAc cannot be regarded as a functional analogue of [^11^C]acetate in terms of HCC evaluation [14]. Rather disappointingly, we also demonstrated that [^18^F]FAc is not favorable for the PET of liver fibrosis. An early study by Lindhe et al. showed that fluorinated compounds could not be easily trapped by ACS1; therefore, [^18^F]FAc was from the bloodstream and excreted to the bile immediately [34]. Collectively, studies that aim to use [^18^F]FAc for potentially evaluating other pathologies in livers should pay careful attention on its metabolism.

Currently available imaging techniques for liver fibrosis assessment include MRI and ultrasound. MR elastography plays a well-defined role in fibrosis estimation [35]; however, its use is not widely available in clinical settings. Moreover, the torso coil used for the abdominal imaging in MRI usually experiences nonuniformity of signals. The signals are only good for superficial structures but attenuate with depth [36]. B-mode ultrasonic imaging [37] and ultrasound elastography [38] are sensitive to tissue stiffness and have been refined in recent years to assess liver fibrosis. Although ultrasound has a high temporal resolution and is viable for use without radiation burden, it offers low spatial resolution and is user dependent. In contrast, PET techniques provide the advantages of good signal homogeneity, few artifacts arising from the bowel gases, and user independence. The information obtained by PET techniques could provide sensitive, comprehensive, and quantitative measurements of hepatic fibrosis in terms of functional imaging information of diseases. Nevertheless, as each imaging modality has its specific significance and limitations, various modalities could be combined, which could allow accurate diagnosis of fibrosis.

The major limitation of this study is that all animals underwent PET scans at 1 and 2 weeks after BDL. The long-term effects of BDL were not explored. Severe liver damage such as cirrhosis was observed 4 weeks after BDL [8]. This study was performed in a BDL-induced liver fibrosis rat model, which might not reflect all types of liver fibrosis. An experiment similar to the current one, but with a long follow-up period, is warranted in future studies. Second, although the IHC results confirmed the increased expression of TSPO after BDL, the levels of macrophages and HSCs were not determined in this study. Previous study has shown that levels of macrophages and HSCs increased after the onset of liver fibrosis [39]. Additional studies using anti-TSPO antibody and anti-CD11b antibody for identifying Kupffer cells and macrophages in the liver are required to further reveal the pathology of liver fibrosis. The last limitation is related to the sample size. Due to the limited number of samples, only IHC staining was performed to confirm the status of liver fibrosis. Western blotting, another widely used analytical technique to detect specific proteins, was not performed in this study. Future studies combining both analytical techniques could provide unambiguous evidence of liver fibrosis detection.

## 5. Conclusion

This study indicated for the first time that liver damage related to fibrosis can be longitudinally monitored using PET techniques based on ^18^F-labeling tracers. Both [^18^F]FEPPA and [^18^F]FDG can serve as sensitive probes for detecting hepatic fibrosis. The negative results of [^18^F]FAc, however, suggest that it is not suitable for use as a potential tracer for hepatic fibrosis detection.

## Figures and Tables

**Table 1:** Comparisons of ALT, ALP, GGT, and albumin measurements.

	Week 0	Week 1	Week 2	*P* value
ALT (U/L)	53 ± 15.1	283.7 ± 38.5	256 ± 38.6	<0.001
ALP (U/L)	95.7 ± 9.1	299.3 ± 59.8	312 ± 41.9	<0.005
GGT (U/L)	13 ± 2.6	35.7 ± 15.3	32.7 ± 2.3	<0.05
Albumin (g/dL)	3.7 ± 0.6	3.5 ± 0.7	3.4 ± 0.3	0.84

^∗^ *P* value from the one-way ANOVA measurement.

## Data Availability

The data can be freely given upon request.

## Supplementary Materials

Supplementary Materials Table S1: the list of abbreviation of Figure 4.
