# Supplementary material for: Comparison of 18F-FDG, 18F-Fluoroacetate, and 18F-FEPPA for Imaging Liver Fibrosis in a Bile Duct-Ligated Rat Model
Source: Mol Imaging. 2021 Nov 27;2021:7545284. doi: 10.1155/2021/7545284 (PMC8654319; doi:10.1155/2021/7545284)
Supplement: Supplementary Materials — Table S1: the list of abbreviation of Figure 4. [file 7545284.f1.docx]

Table S1. The list of abbreviation of Figure 3.

| Collagen Fibril Organization | |
| --- | --- |
| ADAMTS14 | ADAM metallopeptidase with thrombospondin type 1 motif, 14 |
| ADAMTS2 | ADAM metallopeptidase with thrombospondin type 1 motif, 2 |
| ANXA2 | Annexin A2 |
| ATP7A | ATPase copper transporting alpha |
| COL1A1 | Collagen type I alpha 1 chain |
| COL1A2 | Collagen type I alpha 2 chain |
| COL3A1 | Collagen type III alpha 1 chain |
| COL5A1 | Collagen type V alpha 1 chain |
| COL5A2 | Collagen type V alpha 2 chain |
| LOXL2 | Lysyl oxidase-like 2 |
| SERPINH1 | Serpin family H member 1 |
| TGFB2 | Transforming growth factor, beta 2 |
| Extracellular Matrix Organization | |
| APBB1 | Amyloid beta precursor protein binding family B member 1 |
| APP | Amyloid beta precursor protein |
| ATP7A | ATPase copper transporting alpha |
| BCL3 | BCL3, transcription coactivator |
| ELF3 | E74 like ETS transcription factor 3 |
| ELN | Elastin |
| FBLN1 | Elastin microfibril interfacer 1 |
| ITGA8 | Fibulin 1 |
| LGALS3 | Integrin subunit alpha 8 |
| NID1 | Galectin 3 |
| OLFML2A | Nidogen 1 |
| OLFML2B | Olfactomedin-like 2B |
| PXDN | Peroxidasin |
| SMOC2 | SPARC related modular calcium binding 2 |
| SOX9 | SRY-box transcription factor 9 |
| SPINT1 | Serine peptidase inhibitor, Kunitz type 1 |
| TGFB2 | Transforming growth factor, beta 2 |
| TNFRSF11B | TNF receptor superfamily member 11B |
| Metabolism | |
| SLC2A1 | Solute carrier family 2 member 1 |
| SLC2A2 | Solute carrier family 2 member 2 |
| ACSL1 | Acyl-CoA synthetase long-chain family member 1 |
| Inflammation | |
| TSPO | Translocator protein |
